# Investigation into the Synergistic Effect of the Zinc Peroxide/Peroxymonosulfate Double-Oxidation System for the Efficient Degradation of Tetracycline

**DOI:** 10.3390/molecules29174120

**Published:** 2024-08-30

**Authors:** Shefeng Li, Yong Zhang, Siyu Ding, Xuli Li, Wei Wang, Ningning Dong, Miaomiao Nie, Pei Chen

**Affiliations:** 1 School of Chemical and Environmental Engineering, Wuhan Polytechnic University, Wuhan 430023, China; lishefeng@whpu.edu.cn (S.L.); yongzhang@whpu.edu.cn (Y.Z.); siyu_ding@hotmial.com (S.D.); lixuli@whpu.edu.cn (X.L.); 20230312012@whpu.edu.cn (M.N.); 2 Hubei Engineering Research Center for Soil and Groundwater Pollution Control, Wuhan 430070, China; 3 Pilot Base of Ecological Environmental Chemicals and Low-Carbon Technology Transformation, Wuhan 430023, China; 4 State Key Laboratory of Materials Processing and Die & Mould Technology, School of Materials Science and Engineering, Huazhong University of Science and Technology, Wuhan 430074, China; weiwang@hust.edu.cn; 5 Analytical and Testing Center, Huazhong University of Science and Technology, Wuhan 430074, China; 2023310046@hust.edu.cn

**Keywords:** zinc peroxide, peroxymonosulfate, double-oxidation system, tetracycline degradation, synergistic activation mechanism

## Abstract

The increasingly severe antibiotic pollution has become one of the most critical issues. In this study, a zinc peroxide/peroxymonosulfate (ZnO_2_/PMS) double-oxidation system was developed for tetracycline (TC) degradation. A small amount of ZnO_2_ (10 mg) and PMS (30 mg) could effectively degrade 82.8% of TC (100 mL, 50 mg/L), and the degradation process could be well described by the pseudo-second-order kinetic model. Meanwhile, the ZnO_2_/PMS double-oxidation system showed high adaptability in terms of reaction temperature (2–40 °C), initial pH value (4–12), common inorganic anions (Cl^−^, NO_3_^−^, SO_4_^2−^ and HCO_3_^−^), natural water source and organic pollutant type. The quenching experiment and electron paramagnetic resonance (EPR) characterization results confirmed that the main reactive oxygen species (ROS) was singlet oxygen (^1^O_2_). Moreover, three possible pathways of TC degradation were deduced according to the analyses of intermediates. On the basis of comparative characterization and experiment results, a synergistic activation mechanism was further proposed for the ZnO_2_/PMS double-oxidation system, accounting for the superior degradation performance. The released OH^−^ and H_2_O_2_ from ZnO_2_ could activate PMS to produce major ^1^O_2_ and minor superoxide radicals (•O_2_^−^), respectively.

## 1. Introduction

Antibiotics have been widely applied in the fields of clinical treatment, animal husbandry and crop growth [1,2,3,4,5,6]. However, the reckless use of antibiotics results in their frequent detection in water bodies [7,8,9] since the majority of antibiotics are poorly absorbed by organisms or degraded in nature [10]. Residual antibiotics in the environment easily trigger serious environmental problems, like fostering antibiotic resistance [11], inducing physiological toxicity [12] and threatening human health [13]. It has been reported that the concentration of antibiotics in some pig farm wastewater could even reach up to 25 mg/L [14], posing a great need for the efficient treatment of antibiotic-polluted wastewater.

Advanced oxidation processes (AOPs), like photocatalysis [15], ozonation [16], Fenton/Fenton-like oxidation [17] and electrocatalysis [18], have received widespread attention due to the fact that the generated reactive oxygen species (ROS) can degrade and mineralize organic pollutants with high efficiency and environmental friendliness [19,20,21]. Recently, peroxymonosulfate (PMS)-based AOPs have been considered promising and have become one of the research hotspots. This is because a variety of methods, including carbon materials [22], transition metals [23], heat [24], ultraviolet light [25], ultrasound [26] and alkali [27], can facilely activate PMS to degrade organic contaminants. Moreover, the generated ROS contain not only free radicals (like superoxide (•O_2_^–^), hydroxyl (•OH) and sulfate (SO_4_•^–^)) but also non-free radical singlet oxygen (^1^O_2_), which greatly expands the applications of PMS-based AOPs [28].

Among PMS-based AOPs, a hydrogen peroxide (H_2_O_2_)/PMS dual-oxidant system possesses superior performance towards antibiotics degradation. Sun et al. claimed that more than 80% of tetracycline (TC) could be degraded in a biochar/nZVI/MoS_2_-activated H_2_O_2_/PMS double-oxidation system [29]. Under the catalysis of pipe deposits, the H_2_O_2_/PMS double-oxidation system could remove around 80% of chloramphenicol within 120 min [30]. A MnO_2_/UIO-66-activated H_2_O_2_/PMS system could efficiently degrade 79.5% of oxytetracycline with high adaptability towards common inorganic anions [31]. Despite the excellent performance of H_2_O_2_/PMS double-oxidation systems, H_2_O_2_ suffers from the defects of being workable only under acidic conditions and its poor stability [32], severely restricting the practical applications of H_2_O_2_/PMS double-oxidation systems.

Accordingly, some metal peroxides, with relatively high stability and adaptability in a wide pH range, have been developed as an excellent alternative to H_2_O_2_, which can slowly produce H_2_O_2_ by reacting with H_2_O. In addition, the hydrolysis processes of metal peroxides can produce alkaline metal hydroxides, contributing to the activation of PMS. So far, calcium peroxide (CaO_2_) [33,34] has been reported in persulfate-based double-oxidation systems and has exhibited outstanding results in antibiotic degradation. Compared with CaO_2_, zinc peroxide (ZnO_2_) has a lower hydrolysis rate and can release H_2_O_2_ more slowly [35], which is certainly conducive to the effective utilization of H_2_O_2_. This means that ZnO_2_ possesses broad potential in environmental remediation fields. However, ZnO_2_/PMS double-oxidation systems have never been reported in the field of organic pollutant degradation.

In this manuscript, a ZnO_2_/PMS double-oxidation system was developed for the effective degradation of TC. The degradation performance of the ZnO_2_/PMS double-oxidation system was investigated in terms of ZnO_2_ dosage, PMS dosage, reaction temperature and initial solution pH value. Moreover, common inorganic matrices, different natural water sources and various types of organic pollutants were used to assess the adaptability of the ZnO_2_/PMS double-oxidation system. Quenching experiments and electron paramagnetic resonance (EPR) tests were conducted to reveal the main ROS responsible for TC degradation. Furthermore, the possible TC degradation pathways were clarified by an analysis of the intermediate products, and the synergistic effect of the ZnO_2_/PMS double-oxidation system was revealed on the basis of comparative experimental results.

## 2. Experimental Section

### 2.1. Chemicals

Zinc nitrate hexahydrate (Zn(NO_3_)_2_·6H_2_O), hydrogen peroxide (H_2_O_2_), ammonium hydroxide (NH_3_·H_2_O), sodium hydroxide (NaOH), sulfuric acid (H_2_SO_4_), anhydrous ethanol (C_2_H_5_OH), sodium chloride (NaCl), sodium nitrate (NaNO_3_), sodium sulfate (Na_2_SO_4_) and sodium hydrogen carbonate (NaHCO_3_) were purchased from Sinopharm Chemical Reagent Co., Ltd. (Ningbo, China). Tetracycline (C_22_H_24_N_2_O_8_, TC), doxycycline hydrochloride (C_22_H_25_N_2_O_8_Cl, DOXH), Congo red (C_32_H_22_N_6_Na_2_O_6_S_2_, CR), Chloramphenicol hydrochloride (C_22_H_22_Cl_2_N_2_O_7_, CTCH), 5,5-dimethyl-1-pyrroline N-oxide (DMPO), 2,2,6,6-tetramethylpiperidine (TEMP) and 4-hydroxy-2,2,6,6-tetramethylpiperidin-1-oxyl (TEMPOL) were provided by Aladdin Chemistry Reagent Chemistry Co., Ltd. (Shanghai, China). Methanol (CH_3_OH, MeOH), isopropanol (C_3_H_8_O, IPA) and furfuryl alcohol (C_5_H_6_O_2_, FFA) were supplied by Shanghai Chemical Reagent Co., Ltd. (Shanghai, China). All chemicals were of analytical grade and used without further purification.

### 2.2. Synthesis of ZnO_2_ Particles

Briefly, 1.5 mL of H_2_O_2_ was added to 20 mL of Zn(NO_3_)_2_·6H_2_O solution (0.5 mol/L). Then, 1.6 mL of NH_3_·H_2_O was slowly dropped into the above solution under vigorous agitation. In order to investigate the effect of agitation time on the product, a series of preparation experiments were conducted under different agitation times (10 min, 30 min, 60 min and 240 min). After the reaction, the generated white precipitates were separated by centrifugation and washed several times. Finally, the products were reserved after being dried in an oven at 60 °C for 12 h.

### 2.3. Characterizations

The phase structure was determined using an X-ray diffractometer (XRD, x’pert3 powder). The functional group was clarified via a Fourier transform infrared spectrometer (FTIR, Spectrum 3, PerkinElmer, Waltham, MA, USA). The thermal behavior was investigated using a thermogravimetry analyzer (TGA 5500, Milford, MA, USA). The microstructure was characterized by scanning electron microscopy (SEM, Nova NanoSEM 450, FEI, Eindhoven, Netherlands) and transmission electron microscopy (TEM, Tecnai G2 20, FEI, Eindhoven, The Netherlands). The chemical oxygen demand (COD) was tested using a multi-parameter water quality analyzer (LH-T640, Lianhua Technology, Beijing, China), and total organic carbon (TOC) concentration was measured via a TOC analyzer (TOC-L CPH, Shimadzu, Kyoto, Japan). The generated ROS were characterized using an electron paramagnetic resonance (EPR) spectrometer (EMXmicro-6/1, Bruker, Karlsruhe, Germany). The intermediates generated during the TC degradation process were determined using high-resolution liquid chromatography–mass spectrometry (HR-LC-MS, Thermo Fisher Orbitrap Q Exactive, Thermo Fisher Waltham, MA, USA). The specific analysis conditions and parameters are described in Text S1 and Table S1, which are provided in the Supplementary Materials.

### 2.4. Catalytic Degradation Experiment

At the preset temperature (2–40 °C), a certain amount of ZnO_2_ (0–15 mg) and PMS (0–40 mg) were sequentially added to the TC solution (100 mL, 50 mg/L) with different initial pH values (2–12) to start the degradation reaction under continuous agitation. Each group of degradation experiments was performed in triplicate. At specific time intervals, 3 mL of the mixture was extracted and filtered through a 0.22 μm filter. The residual TC concentration was measured by a UV-Vis spectrometer (UV-8000S), and the degradation rate was calculated using Equation (1).

Degradation rate (%) = (1 − C_t_/C_o_) × 100% (1)

where C_o_ and C_t_ (mg/L) were TC concentrations at initial and certain times (t), respectively.

## 3. Results and Discussion

### 3.1. Characterizations of ZnO_2_

**Figure 1:**
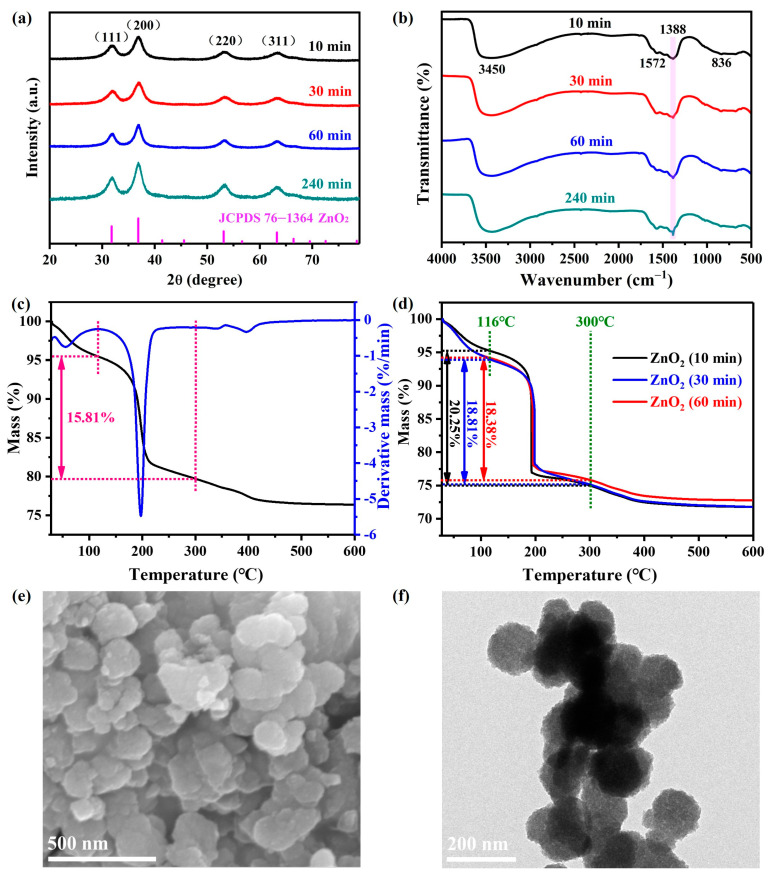
XRD spectra (**a**) and FTIR patterns (**b**) of the products under different conditions; TG plots of ZnO_2_ samples under agitation for 240 min (**c**) and other agitation times (**d**); SEM (**e**) and TEM (**f**) pictures of as-synthesized ZnO_2_ under agitation for 240 min.

The XRD patterns of as-synthesized ZnO_2_ particles under different agitation times are displayed in Figure 1a. Evidently, these four characteristic diffraction peaks centered at 31.9°, 36.9°, 53.4° and 63.3° could be well assigned to the (111), (200), (220) and (311) planes of ZnO_2_ with the standard cubic phase (JCPDS 76−1364), respectively. Furthermore, the intensity of the diffraction peaks was enhanced with the agitation time, which indicated that extending the agitation time could be well conducive to the crystallinity. According to the Scherrer equation, the crystallite size of ZnO_2_ under agitation for 240 min was calculated to be 10.72 nm (Table S2), which was consistent with an earlier reported study [36]. As for the FTIR spectra of ZnO_2_ (Figure 1b), the absorption peaks around 3450 and 1572 cm^−1^ were attributed to -OH vibration [37]. In addition, the two adsorption peaks located at 1388 and 849 cm^−1^ were assigned to the characteristic O-O vibration [9]. It could be clearly observed that the intensity of the adsorption peak at 1388 improved with the agitation time. This point confirmed that extending the agitation time contributed to the formation of the O-O bond, which is consistent with the XRD results.

Figure 1c displays the thermal behavior of ZnO_2_ under agitation for 240 min. It could be observed that around 4.5% of the mass was lost from 30 to 116 °C, which is attributed to the desorption of adsorbed water. The main mass loss (15.81%) rapidly occurred between 116 and 300 °C, which was attributed to the thermal decomposition of ZnO_2_. Thus, the purity could be calculated to be around 96.4% in view of the theoretical value (16.4%). Meanwhile, the mass losses of ZnO_2_ samples under agitation for 10 min, 30 min and 60 min at this stage were 20.25, 18.81 and 18.38%, respectively (Figure 1d). They were all higher than the theoretical value, which indicated the generation of amorphous impurities due to incomplete oxidation. Accordingly, the ZnO_2_ sample prepared after agitation for 240 min was chosen for subsequent characterizations and degradation experiments.

The microscopic morphology of as-prepared ZnO_2_ was characterized by SEM and TEM techniques. As shown in Figure 1e, the as-prepared ZnO_2_ was mainly composed of multiple dispersive spherules with a particle size of around 100 nm. As seen from the TEM image (Figure 1f), the spherules were indeed assembled from much smaller primary particles, which was verified by the crystallite size calculation results. Undoubtedly, the highly dispersed particles would tend to expose active sites, thus contributing to the catalytic activity.

### 3.2. Degradation Performance of ZnO_2_/PMS Double-Oxidation System

#### 3.2.1. Effects of PMS and ZnO_2_ Dosages

**Figure 2:**
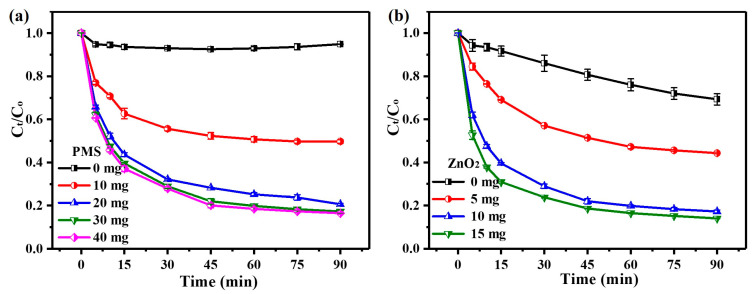
Effects of PMS dosage (**a**) and ZnO_2_ dosage (**b**) on TC degradation.

In order to investigate the effect of PMS dosage on the performance of the ZnO_2_/PMS double-oxidation system, the ZnO_2_ dosage was fixed at 10 mg. As shown in Figure 2a, only around 5% of TC was removed without PMS addition, which can be attributed to the physical adsorption of ZnO_2_. After PMS addition, the degradation rate of TC increased with the PMS dosage. After 60 min, the degradation rate of TC rose significantly from 49.3% to 80.2% when the dosage of PMS was increased from 10 mg to 30 mg. However, the degradation rate was slightly enhanced as the PMS dosage was further increased to 40 mg. In consideration of the reagent cost and degradation efficiency, 30 mg of PMS was selected for the ZnO_2_/PMS double-oxidation system in subsequent degradation experiments.

The influence of the ZnO_2_ dosage is displayed in Figure 2b. It can be clearly observed that nearly 31% of TC was degraded within 90 min by PMS alone. After the addition of a small dosage of ZnO_2_ (5 mg), the degradation rate improved remarkably to 55.7% after 90 min, indicating the significant role of ZnO_2_ in the degradation system. It was noted that a slight enhancement of the degradation rate (only 3.2%) was realized after increasing the ZnO_2_ dosage from 10 to 15 mg. Hence, 10 mg of ZnO_2_ was deemed suitable for the ZnO_2_/PMS double-oxidation system in subsequent degradation experiments. Meanwhile, pseudo-zero-order, pseudo-first-order and pseudo-second-order kinetic models (Equations (S1)–(S3)) were employed to explore the kinetic characteristics of TC degradation (Text S2). The results (Figure S1) indicate that the pseudo-second-order kinetic model was more suitable to describe TC degradation on the basis of linear correlation coefficients, as listed in Table S3. This point illustrated that the degradation rate of TC was positively correlated with the square of dominant ROS concentrations generated in the ZnO_2_/PMS double-oxidation system.

#### 3.2.2. Effects of Temperature and Initial Solution pH

**Figure 3:**
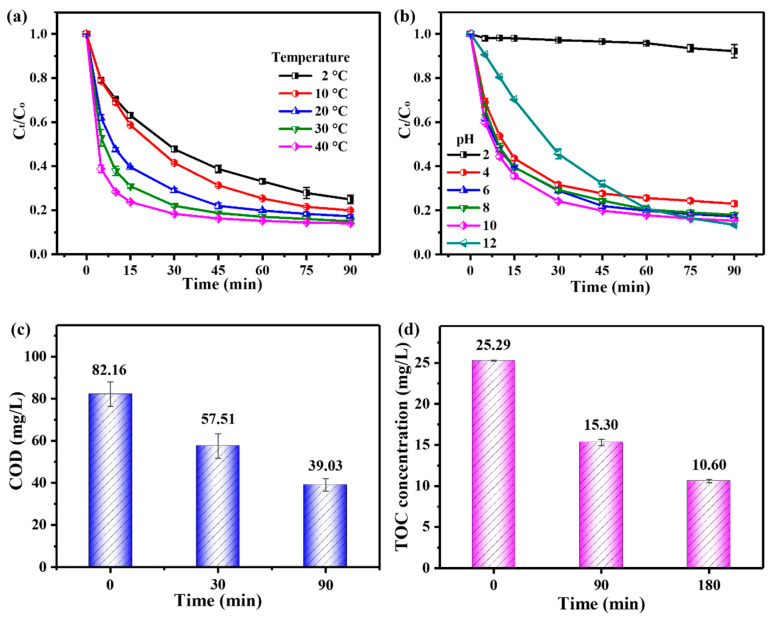
Effects of temperature (**a**) and initial solution pH (**b**) on TC degradation; COD (**c**) and TOC (**d**) removal in the ZnO_2_/PMS double-oxidation system.

The performance of the ZnO_2_/PMS double-oxidation system for TC degradation was examined under different temperatures (2–40 °C). The results (Figure 3a) exhibit that over 75% of TC could be degraded even at a low temperature (2 °C), revealing the terrific degradation performance of the ZnO_2_/PMS double-oxidation system. The degradation rate was continuously enhanced with the rise in temperature, and the degradation rate reached up to 80% within 60 min at 20 °C. Considering the extent of improvement became quite weak as the temperature exceeded 20 °C, the subsequent degradation experiments were conducted under 20 °C.

To explore the effect of initial pH, the degradation experiments were performed with different initial pH values (2–12). As seen from Figure 3b, under strong acid conditions (pH = 2), only 7.8% of TC was degraded after 90 min. This was due to the fact that ZnO_2_ had been completely decomposed by the large numbers of hydrogen ions (H^+^) in the solution. The generation of ROS via the self-decomposition of PMS was greatly inhibited under strong acid conditions [38], resulting in the low degradation rate of TC. Notably, the degradation rate was significantly improved with the increase in the initial solution pH, and around 77% of TC could be effectively degraded when the initial solution pH was adjusted to 4. It could be found that the ZnO_2_/PMS double-oxidation system showed excellent performance for TC degradation in a wide initial pH range (4–12).

Moreover, the Arrhenius equation (Equation (S4)) was applied to calculate the reaction activation energy (Text S3). As illustrated in Figure S2, the positive activation energy (32.3 KJ/mol) indicated that the TC degradation process was endothermic and in agreement with the contribution of increasing temperature to the degradation performance of the ZnO_2_/PMS double-oxidation system. Figure 3c exhibits the change in COD during the TC degradation process. It can be observed that the COD value significantly dropped from an initial 82.16 to 39.03 mg/L after 90 min, and the COD removal efficiency was over 52%. The TOC removal results (Figure 3d) show that the TOC concentration declined from 25.29 to 10.60 mg/L. Over 58% of TOC was removed after 180 min, suggesting a high mineralization efficiency of the ZnO_2_/PMS double-oxidation system in TC degradation.

#### 3.2.3. Adaptability of ZnO_2_/PMS Double-Oxidation System

**Figure 4:**
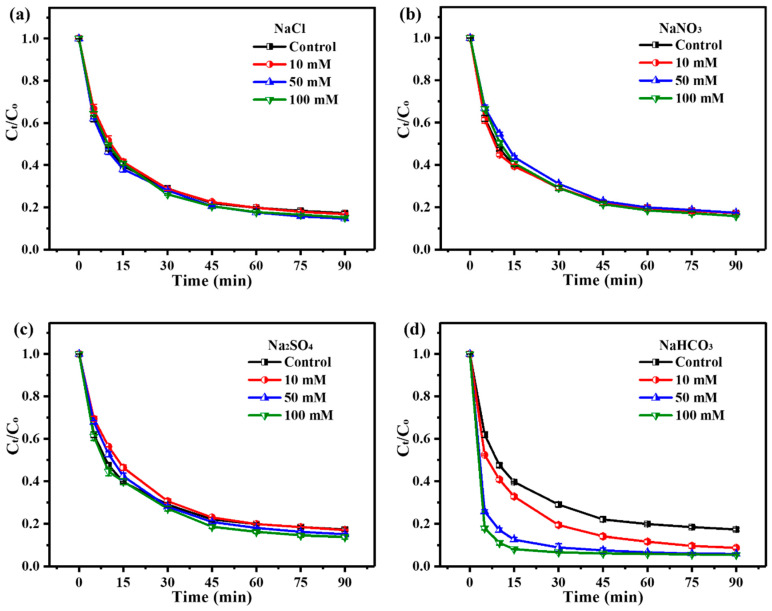
Adaptability experiments of ZnO_2_ + PMS system to Cl^−^ (**a**), NO_3_^−^ (**b**), SO_4_^2−^ (**c**) and HCO_3_^−^ (**d**).

Firstly, the adaptability of the ZnO_2_/PMS double-oxidation system was evaluated by four common matrix species (NaCl, NaNO_3_, Na_2_SO_4_ and NaHCO_3_) with different concentrations (10–100 mM). As shown in Figure 4a–c, NaCl, NaNO_3_ and Na_2_SO_4_ could rarely affect TC degradation. Surprisingly, the existence of NaHCO_3_ remarkably enhanced TC degradation (Figure 4d), and this promotion effect could be attributed to the improvement of the solution pH value after NaHCO_3_’s addition (Figure S3a), which was consistent with the effect of the initial solution pH on TC degradation (Figure 3b).

**Figure 5:**
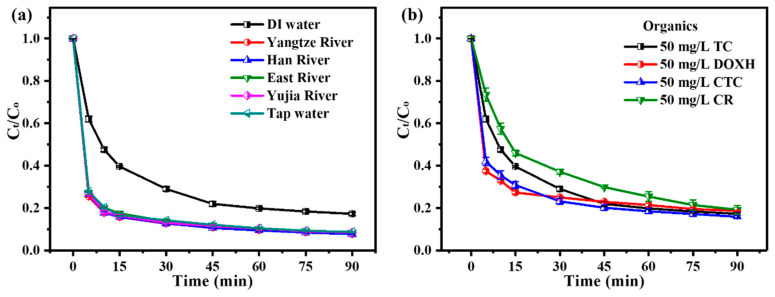
Adaptability experiments of ZnO_2_ + PMS system to natural water resources (**a**) and organic pollutant types (**b**).

Then, several natural water resources (Yangtze River water, Han River water, East Lake water, Yujia Lake water and tap water) were studied to explore the adaptability of the ZnO_2_/PMS double-oxidation system. The specific water quality parameters of these natural water resources are listed in Table S4. Unexpectedly, the results (Figure 5a) show that TC degradation was enhanced in these natural water resources. This could be due to the higher initial pH values of the TC solution when prepared using natural water resources (Figure S3b).

Additionally, diverse kinds of organic pollutants (DOXH, CR and CTCH) were used to investigate the adaptability of the ZnO_2_/PMS double-oxidation system. As displayed in Figure 5b, the degradation rates of DOXH, CR and CTCH could reach 81.2%, 80.1% and 84.1% after 90 min, respectively. On the basis of the above results, it was confirmed that the ZnO_2_/PMS double-oxidation system possessed high adaptability to common matrix species, water resources and organic pollutant types.

### 3.3. Mechanism Analysis

#### 3.3.1. Quenching Experiments

**Figure 6:**
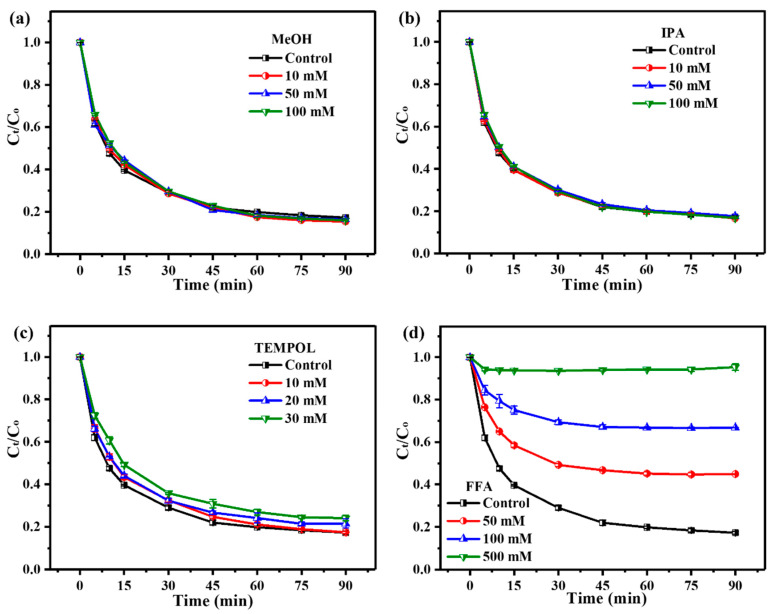
Quenching experiments with different concentrations of MeOH (**a**), IPA (**b**), TEMPOL (**c**) and FFA (**d**).

Quenching experiments were conducted to explore the generated ROS in the ZnO_2_/PMS double-oxidation system. Specifically, MeOH, IPA and FFA were selected to quench the possible SO_4_•^−^, •OH and ^1^O_2_ generated in the system, respectively. Due to the fact that *p*-BQ can activate PMS to produce non-radical ^1^O_2_ [39], TEMPOL was utilized to quench generated •O_2_^−^ radicals as a substitute for *p*-BQ [40]. As displayed in Figure 6a,b, the addition of MeOH and IPA with different concentrations (10–100 mM) had little effect on TC degradation, which indicated that the ZnO_2_/PMS double-oxidation system could hardly produce SO_4_•^−^ and •OH radicals.

As seen from Figure 6c, there was a slight inhibition effect, and the degradation rate only decreased by 5% after 30 mM TEMPOL was added into the system, suggesting the existence of a small amount of •O_2_^−^ radicals. As for FFA (Figure 6d), it could significantly inhibit TC degradation, and the degradation rate dropped from 82.8% to 55.1% after the addition of 50 mM FFA. As the concentration of FFA reached 500 mM, TC degradation was completely inhibited in the system. Accordingly, the above results indicated that the main ROS was ^1^O_2_, accompanied by a small amount of •O_2_^−^.

**Figure 7:**
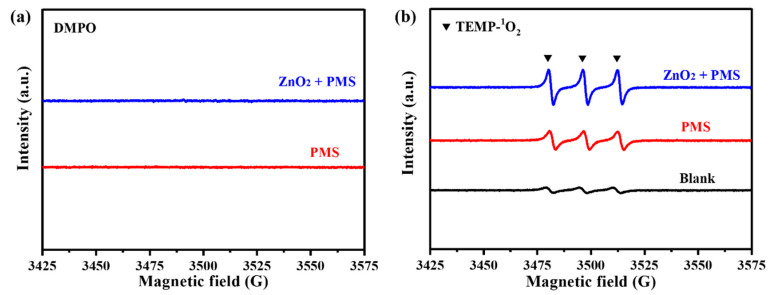
EPR tests of ZnO_2_ + PMS system using DMPO (**a**) and TEMP (**b**).

Further, EPR tests were carried out to identify the ROS generated in the ZnO_2_/PMS double-oxidation system. It was noted that SO_4_•^−^, •OH and •O_2_^−^ radicals could react with DMPO to form characteristic signals. As shown in Figure 7a, no obvious signals could be detected in the ZnO_2_ + PMS system. This point demonstrated that the ZnO_2_ + PMS system could not produce SO_4_•^−^ and •OH radicals, which is consistent with the quenching experiment results (Figure 6a,b). The characteristic signals related to the DMPO-•O_2_^−^ adduct were not detected either, which might be attributed to the low number of •O_2_^−^ radicals. The EPR results using TEMP as a capturing agent are exhibited in Figure 7b. Clearly, the characteristic triplet signal of the TEMP-^1^O_2_ adduct with an intensity ratio of 1:1:1 could be observed in the PMS system because PMS could generate ^1^O_2_ through a self-decomposition reaction [41]. As for the ZnO_2_ + PMS system, the signal intensity of TEMP-^1^O_2_ was much stronger than that in the sole PMS system. This point confirmed that ZnO_2_ can activate PMS to produce more ^1^O_2_, thereby contributing to the superior performance for TC degradation compared with other PMS-based systems (Table 1).

#### 3.3.2. Intermediate Identification and TC Degradation Pathway Investigation

**Figure 8:**
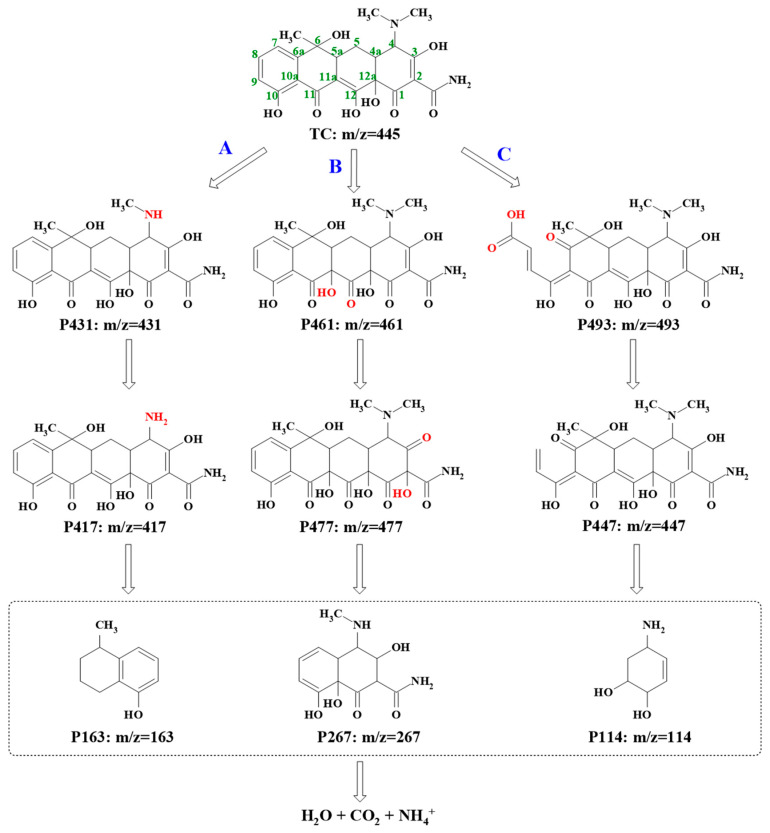
Possible pathways for TC degradation in ZnO_2_/PMS double-oxidation system.

To deduce the possible pathways of TC degradation in the ZnO_2_/PMS double-oxidation system, the HR-LC-MS technique was employed to determine the generated intermediates. As shown in the ESI-MS spectra (Figure S4), TC (P445) and nine main intermediates (P493, P449, P461, P477, P431, P417, P267, P163 and P114) were found at different retention times and the detailed information on this is supplied in Table S5. Based on the detected intermediates, three possible degradation pathways are proposed in Figure 8. In pathway A, a TC molecule first lost a methyl group around C4 to generate P431 [48] and formed P417 through further demethylation. In pathway B, the product of P461 was first generated through dipolar cycloaddition and the rearrangement of the hydroxyl group at C11a-C12 in the TC molecule. P461 progressively transformed into P477 via hydroxylation [49]. As for pathway C, the TC molecule opened the ring at C7 to produce P493 [50]. Then, P447 was produced via the decarboxylation of P493 at C8. Subsequently, the smaller intermediates, including P163, P267 and P114, were produced by further oxidation [51]. Finally, these intermediates were degraded and mineralized into CO_2_, H_2_O and NH_4_^+^.

#### 3.3.3. Activation Pathway Study

**Figure 9:**
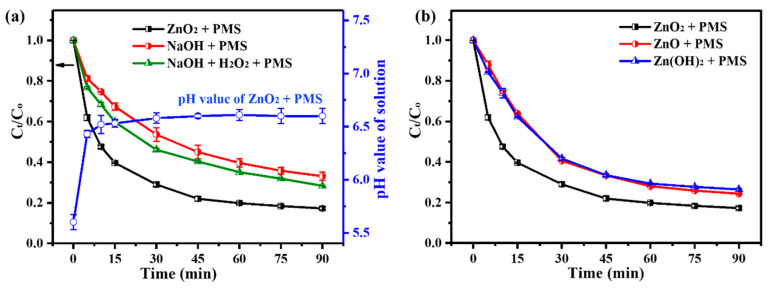
Comparison of degradation performance of NaOH + PMS, NaOH + H_2_O_2_ + PMS and ZnO_2_ + PMS systems under the same pH conditions (**a**), and using equimolar amount of ZnO, Zn(OH)_2_ and ZnO_2_ to activate PMS for TC degradation (**b**).

It was well known that hydroxide ions (OH^−^) could activate PMS to generate ^1^O_2_ [52,53] and that Zn(OH)_2_ released from ZnO_2_ would spontaneously produce OH^−^ in solution via the ionization effect. Thus, it is of great significance to monitor the pH value of the ZnO_2_/PMS double-oxidation system during TC degradation in order to deeply understand the generation mechanism of ROS in the ZnO_2_/PMS double-oxidation system. As illustrated in Figure 9a, the pH value of the ZnO_2_/PMS double-oxidation degradation system (ZnO_2_ + PMS) increased from the initial 5.6 to 6.4 and gradually stabilized at around 6.6.

Then, two comparative degradation experiments (NaOH + PMS and NaOH + H_2_O_2_ + PMS) were conducted under the same pH condition as the ZnO_2_ + PMS system, and the molar amount of H_2_O_2_ equaled the molar amount of ZnO_2_. The results showed that the degradation rate of TC in the NaOH + PMS system reached 67% within 90 min, proving that OH^−^ could indeed activate PMS to produce ^1^O_2_. As for the NaOH + H_2_O_2_ + PMS system, on one hand, TC degradation was apparently enhanced in comparison with the NaOH + PMS system, demonstrating that PMS might be activated by H_2_O_2_. It was reported that H_2_O_2_ could activate PMS to produce •O_2_^−^ radicals [54], which was consistent with the TEMPOL quenching experiment results (Figure 6c). On the other hand, the degradation performance of the NaOH + H_2_O_2_ + PMS system was still inferior to the ZnO_2_ + PMS system, revealing the contribution of ZnO_2_ release to the TC degradation in the ZnO_2_/PMS double-oxidation system.

Additionally, equimolar ZnO and Zn(OH)_2_ were selected to replace ZnO_2_ for PMS activation for TC degradation under the same conditions. The results (Figure 9b) showed that both the ZnO + PMS system and the Zn(OH)_2_ + PMS system had an inferior performance in TC degradation. This point confirmed that the released H_2_O_2_ from ZnO_2_ was indeed able to activate PMS, thereby promoting the degradation performance of the ZnO_2_/PMS double-oxidation degradation system.

Based on the above results and analyses, the generation mechanism of ROS in the ZnO_2_/PMS double-oxidation system could be deduced by the following equations (Equations (2)–(8)). It was well known that PMS could directly produce ^1^O_2_ through the self-decomposition reaction (Equation (2)), according to previous research [55]. In addition, as illustrated in Equation (3), ZnO_2_ would transform into Zn(OH)_2_ and H_2_O_2_ via hydrolysis in solution [36]. On the one hand, the generated Zn(OH)_2_ could spontaneously release OH^−^ ions (Equation (4)). It was reported that OH^−^ could activate PMS to generate ^1^O_2_ via Equation (5) [27], thus contributing to TC degradation. On the other hand, the ionization reaction of the produced H_2_O_2_ (Equation (6)) was greatly promoted under alkaline conditions, which was revealed in the base-activated PMS system [56]. Further, the ionization product HO_2_^−^ would react with PMS to form •O_2_^−^ radicals [33] through Equation (7). Therefore, TC was effectively degraded by the ^1^O_2_ and •O_2_^−^ produced in the ZnO_2_/PMS double-oxidation system (Equation (8)).

HSO_5_^−^ + SO_5_^2−^ → HSO_4_^−^ + SO_4_^2−^ + ^1^O_2_ (2)

ZnO_2_ + 2H_2_O → Zn(OH)_2_ + H_2_O_2_ (3)

Zn(OH)_2_ → Zn^2+^ + 2OH^−^ (4)

HSO_5_^−^ + SO_5_^2−^ + OH^−^ → 2SO_4_^2−^ + H_2_O + ^1^O_2_ (5)

H_2_O_2_ → HO_2_^−^ + H^+^ (6)

2SO_5_^2−^ + HO_2_^−^ → 2SO_4_^2−^ + 2•O_2_^−^ + H^+^ (7)

^1^O_2_ + •O_2_^−^ + TC → Intermediate products + H_2_O + CO_2_ + NH_4_^+^ (8)

## 4. Conclusions

In summary, a ZnO_2_/PMS double-oxidation system was fabricated and applied to the degradation of organic pollutants. Over 80% of 50 mg/L TC could be effectively degraded by 0.1 g/L ZnO_2_ and 0.3 g/L PMS within 60 min. Surprisingly, the ZnO_2_/PMS double-oxidation system possessed a relatively strong anti-interference ability towards co-existing inorganic ions, natural water sources and organic pollutant types, exhibiting a great application potential for practical wastewater treatment. ^1^O_2_ was considered the dominant ROS responsible for TC degradation. According to a series of characterization analyses and comparative experiment results, OH^−^ and H_2_O_2_ released from ZnO_2_ were able to activate PMS to generate ^1^O_2_ and minor •O_2_^−^, respectively. It was the existence of a synergistic activation mechanism that endowed the ZnO_2_/PMS double-oxidation system with a superior degradation performance.

## Figures and Tables

**Table 1:** Comparison of performances over the reported PMS-based systems.

PMS-Based Systems	PMS Dosage (g/L)	Catalyst Dosage (g/L)	Concentration of TC (mg/L)	Reaction Time (min)	Degradation Rate (%)	Reference
ZnO_2_ + PMS	0.3	0.1	50	60	80.2	This work
Goethite/biochar composite + PMS	0.62	0.1	30	60	~73%	[42]
MoS_2_/biochar + PMS	0.62	0.5	20	120	78%	[43]
Piggery sludge-derived magnetic biochar + PMS	0.2	0.5	10	120	77.23%	[44]
Ferromanganese oxide + PMS	0.4	0.4	50	80	~79%	[45]
Mn(II)-doped MoS_2_@ alumina + PMS + light	0.4	1.9	20	60	82.4	[46]
ZIF-8/PAN-derived porous carbon + PMS	0.5	0.5	50	120	85.1%	[47]

## Data Availability

The original contributions presented in the study are included in the article and Supplementary Materials, further inquiries can be directed to the corresponding author.

## Supplementary Materials

The following supporting information can be downloaded at: https://www.mdpi.com/article/10.3390/molecules29174120/s1, Text S1. HR-LC-MS analysis conditions and parameters; Table S1. Gradient elution program; Table S2. Crystallite sizes of ZnO2 samples prepared under different agitation time; Text S2. Degradation kinetics analysis; Figure S1. Linear curves fitted by pseudo-zero order (a), pseudo-first order (b) and pseudo-second order (c) kinetic models; Table S3. Kinetic parameters of different kinetics models; Text S3. Degradation thermodynamics analysis; Figure S2. Linear curves fitted by Arrhenius equation; Figure S3. Initial pH values of TC solutions containing common inorganic anions (a) and prepared using different water sources (b); Table S4. Water quality parameters of natural water resources; Figure S4. The detected mass spectra of TC and intermediates; Table S5. Characteristics of intermediate products during TC degradation.

## References

[1] Liu J., Dong Y., Liu Q., Liu W., Lin H. (2024). MoS_2_-based nanocomposites and aerogels for antibiotic pollutants removal from wastewater by photocatalytic degradation process: A review. Chemosphere.

[2] Shan Y., Liu Y., Feng L., Yang S., Tan X., Liu Z. (2024). Magnetic Fe_3_O_4_-C@MoS_2_ composites coordinated with peroxymonosulfate catalysis for enhanced tetracycline degradation. J. Alloys Compd..

[3] Zhao H., He T., Zhang Q., Feng H., Mi H., Liang Z., Zhou D., Dong W., Xue X. (2024). Interfacial bonded K-doped-C_3_N_4_@Bi_2_WO_6_ heterostructure for efficient photocatalytic degradation of tetracycline. J. Alloys Compd..

[4] Xu X., Shao W., Tai G., Yu M., Han X., Han J., Wu G., Xing W. (2024). Single-atomic Co-N site modulated exciton dissociation and charge transfer on covalent organic frameworks for efficient antibiotics degradation via peroxymonosulfate activation. Sep. Purif. Technol..

[5] Wang Z., Xiang M., Huo B., Wang J., Yang L., Ma W., Qi J., Wang Y., Zhu Z., Meng F. (2023). A novel ZnO/CQDs/PVDF piezoelectric system for efficiently degradation of antibiotics by using water flow energy in pipeline: Performance and mechanism. Nano Energy.

[6] Goudarzi M., Abdulhusain Z.H., Salavati-Niasari M. (2023). Low-cost and eco-friendly synthesis of Mn-doped Tl_2_WO_4_ nanostructures for efficient visible light photocatalytic degradation of antibiotics in water. Sol. Energy.

[7] Wang Y., Dong X., Zang J., Zhao X., Jiang F., Jiang L., Xiong C., Wang N., Fu C. (2023). Antibiotic residues of drinking-water and its human exposure risk assessment in rural Eastern China. Water Res..

[8] Yu X., Yu F., Li Z., Zhan J. (2023). Occurrence, distribution, and ecological risk assessment of pharmaceuticals and personal care products in the surface water of the middle and lower reaches of the Yellow River (Henan section). J. Hazard. Mater..

[9] Chen P., Dong N., Zhang J., Wang W., Tan F., Wang X., Qiao X., Wong P.K. (2022). Investigation on visible-light photocatalytic performance and mechanism of zinc peroxide for tetracycline degradation and Escherichia coli inactivation. J. Colloid Interface Sci..

[10] Li S., Zhu L. (2023). Copper regulates degradation of typical antibiotics by microalgal-fungal consortium in simulated swine wastewater: Insights into metabolic routes and dissolved organic matters. Water Res..

[11] Fang S., Huang Y., Xiang Z., Zeng R., Zeng S., Liu S. (2023). Polystyrene nanoplastics foster Escherichia coli O157, H7 growth and antibiotic resistance with a stimulating effect on metabolism. Environ. Sci. Nano.

[12] Zhang S., Han W., Liu T., Feng C., Jiang Q., Zhang B., Chen Y., Zhang Y. (2024). Tetracycline inhibits the nitrogen fixation ability of soybean (*Glycine max* (L.) Merr.) nodules in black soil by altering the root and rhizosphere bacterial communities. Sci. Total Environ..

[13] Míguez-González A., Cela-Dablanca R., Barreiro A., Rodríguez-López L., Rodríguez-Seijo A., Arias-Estévez M., Núñez-Delgado A., Fernández-Sanjurjo M.J., Castillo-Ramos V., Álvarez-Rodríguez E. (2023). Adsorption of antibiotics on bio-adsorbents derived from the forestry and agro-food industries. Environ. Res..

[14] Zhi S., Shen S., Zhou J., Ding G., Zhang K. (2020). Systematic analysis of occurrence, density and ecological risks of 45 veterinary antibiotics: Focused on family livestock farms in Erhai Lake basin, Yunnan, China. Environ. Pollut..

[15] Ali H., Masar M., Yasir M., Machovsky M., Monteiro O.C., Kuritka I. (2023). Current trends in environmental and energy photocatalysis and ISO standardization. J. Environ. Chem. Eng..

[16] Mutke X.A.M., Swiderski P., Drees F., Akin O., Lutze H.V., Schmidt T.C. (2024). Efficiency of ozonation and sulfate radical-AOP for removal of pharmaceuticals, corrosion inhibitors, X-ray contrast media and perfluorinated compounds from reverse osmosis concentrates. Water Res..

[17] Yang Y., Liu M., You X., Li Y., Lin H., Chen J.P. (2024). A novel bimetallic Fe-Cu-CNT catalyst for effective catalytic wet peroxide oxidation: Reaction optimization and mechanism investigation. Chem. Eng. J..

[18] Zeng J., Xie W., Guo Y., Zhao T., Zhou H., Wang Q., Li H., Guo Z., Xu B.B., Gu H. (2024). Magnetic field facilitated electrocatalytic degradation of tetracycline in wastewater by magnetic porous carbonized phthalonitrile resin. Appl. Catal. B Environ..

[19] Xie Z.H., He C.S., Zhou H.Y., Li L.L., Liu Y., Du Y., Liu W., Mu Y., Lai B. (2022). Effects of molecular structure on organic contaminants’ degradation efficiency and dominant ROS in the advanced oxidation process with multiple ROS. Environ. Sci. Technol..

[20] Zhang W., Zhang S., Meng C., Zhang Z. (2023). Nanoconfined catalytic membranes assembled by cobalt-functionalized graphitic carbon nitride nanosheets for rapid degradation of pollutants. Appl. Catal. B Environ..

[21] Tang R., Zeng H., Deng Y., Xiong S., Li L., Zhou Z., Wang J., Tang L. (2023). Dual modulation on peroxymonosulfate activation site and photocarrier separation in carbon nitride for efficient photocatalytic organics degradation: Efficacy and mechanism evaluation. Appl. Catal. B Environ..

[22] Kohantorabi M., Moussavi G., Giannakis S. (2021). A review of the innovations in metal-and carbon-based catalysts explored for heterogeneous peroxymonosulfate (PMS) activation, with focus on radical vs. non-radical degradation pathways of organic contaminants. Chem. Eng. J..

[23] Peng Y., Tang H., Yao B., Gao X., Yang X., Zhou Y. (2021). Activation of peroxymonosulfate (PMS) by spinel ferrite and their composites in degradation of organic pollutants: A Review. Chem. Eng. J..

[24] Ahn Y.Y., Choi J., Kim M., Kim M.S., Lee D., Bang W.H., Yun E.T., Lee H., Lee J.H., Lee C. (2021). Chloride-mediated enhancement in heat-induced activation of peroxymonosulfate: New reaction pathways for oxidizing radical production. Environ. Sci. Technol..

[25] Alayande A.B., Hong S. (2022). Ultraviolet light-activated peroxymonosulfate (UV/PMS) system for humic acid mineralization: Effects of ionic matrix and feasible application in seawater reverse osmosis desalination. Environ. Pollut..

[26] Lee Y., Lee S., Cui M., Ren Y., Park B., Ma J., Han Z., Khim J. (2021). Activation of peroxodisulfate and peroxymonosulfate by ultrasound with different frequencies: Impact on ibuprofen removal efficient, cost estimation and energy analysis. Chem. Eng. J..

[27] Wang W., Chen M., Wang D., Yan M., Liu Z. (2021). Different activation methods in sulfate radical-based oxidation for organic pollutants degradation: Catalytic mechanism and toxicity assessment of degradation intermediates. Sci. Total Environ..

[28] Gao Y., Wu T., Yang C., Ma C., Zhao Z., Wu Z., Cao S., Geng W., Wang Y., Yao Y. (2021). Activity trends and mechanisms in peroxymonosulfate-assisted catalytic production of singlet oxygen over atomic metal-N-C catalysts. Angew. Chem. Int. Ed..

[29] Sun H., Zhang B., Wang N., Zhang N., Ma Y., Zang L., Li Z., Xue R. (2023). Refractory organics removal in PMS and H_2_O_2_/PMS oxidation system activated by biochar/nZVI/MoS_2_ composite: Synthesis, performance, mechanism and dosing methods. J. Environ. Chem. Eng..

[30] Zhong D., Zhou Z., Ma W., Ma J., Lv W., Feng W., Du X., He F. (2022). Study on degradation of chloramphenicol by H_2_O_2_/PMS double-oxidation system catalyzed by pipe deposits from water networks. J. Environ. Chem. Eng..

[31] Zhou Y., Feng S., Duan X., Zheng W., Shao C., Wu W., Jiang Z., Lai W. (2021). MnO_2_/UIO-66 improves the catalysed degradation of oxytetracycline under UV/H_2_O_2_/PMS system. J. Solid State Chem..

[32] Luo Y., Zhang B., Liu C., Xia D., Ou X., Cai Y., Zhou Y., Jiang J., Han B. (2023). Sulfone-Modified Covalent Organic Frameworks Enabling Efficient Photocatalytic Hydrogen Peroxide Generation via One-Step Two-Electron O_2_ Reduction. Angew. Chem. Int. Ed..

[33] Hou Z., Wang W., Dong N., Chen P., Ge L., Tan F., Wang X., Qiao X., Wong P.K. (2023). A dual-oxidant advanced oxidation process system containing CaO_2_ and peroxymonosulfate for organic pollutant degradation: High adaptability and synergistic effect. Sep. Purif. Technol..

[34] Abbas Q., Shakoor A., Naushad M., Naushad M., Yousaf B. (2022). In-situ oxidative degradation of sulfamethoxazole by calcium peroxide/persulfate dual oxidant system in water and soil. Process Saf. Environ. Prot..

[35] Wolanov Y., Prikhodchenko P.V., Medvedev A.V., Pedahzur R., Lev O. (2013). Zinc dioxide nanoparticulates: A hydrogen peroxide source at moderate pH. Environ. Sci. Technol..

[36] Chen P., Wang W., Dong N., Zhang J., Yang T., Tan F., Tan S., Wang X., Qiao X., Wong P.K. (2022). Facile in-situ fabrication of ZnO_2_/CQD composites with promoted visible-light photocatalytic activities for organic degradation and bacterial inactivation. Appl. Surf. Sci..

[37] Wang B., Hu J., Liu K., Zhang L., Jiang H., Li C. (2023). Reinforcement mechanism of silica surface hydroxyl: The opposite effect. Appl. Surf. Sci..

[38] Nie Y., Zhou H., Tian S., Tian X., Yang C., Li Y., Tian Y., Wang Y. (2022). Anionic ligands driven efficient ofloxacin degradation over LaMnO_3_ suspended particles in water due to the enhanced peroxymonosulfate activation. Chem. Eng. J..

[39] Zhang H., Yu K., He J., Li N., You H., Jiang J. (2018). Droplet spray ionization mass spectrometry for real-time monitoring of activation of peroxymonosulfate by 1,4-benzoquinone. Microchem. J..

[40] Liang S., Zheng W., Zhu L., Duan W., Wei C., Feng C. (2019). One-Step treatment of phosphite-laden wastewater: A single electrochemical reactor integrating superoxide radical-induced oxidation and electrocoagulation. Environ. Sci. Technol..

[41] Liu L., Li Y., Li W., Zhong R., Lan Y., Guo J. (2020). The efficient degradation of sulfisoxazole by singlet oxygen (^1^O_2_) derived from activated peroxymonosulfate (PMS) with Co_3_O_4_–SnO_2_/RSBC. Environ. Res..

[42] Guo Y., Yan L., Li X., Yan T., Song W., Hou T., Tong C., Mu J., Xu M. (2021). Goethite/biochar-activated peroxymonosulfate enhances tetracycline degradation: Inherent roles of radical and non-radical processes. Sci. Total Environ..

[43] Su X., Guo Y., Yan L., Wang Q., Zhang W., Li X., Song W., Li Y., Liu G. (2022). MoS_2_ nanosheets vertically aligned on biochar as a robust peroxymonosulfate activator for removal of tetracycline. Sep. Purif. Technol..

[44] Luo X., Shen M., Liu J., Ma Y., Gong B., Liu H., Huang Z. (2021). Resource utilization of piggery sludge to prepare recyclable magnetic biochar for highly efficient degradation of tetracycline through peroxymonosulfate activation. J. Clean. Prod..

[45] Yang Q., Yang X., Yan Y., Sun C., Wu H., He J., Wang D. (2018). Heterogeneous activation of peroxymonosulfate by different ferromanganese oxides for tetracycline degradation: Structure dependence and catalytic mechanism. Chem. Eng. J..

[46] Zhang H., Liu C., Wang Y., Jia F., Song S. (2022). Construction of 3D-sized Mn (II)-doped MoS_2_@activated alumina beads as PMS activator for tetracycline degradation under light irradiation. Chem. Phys. Lett..

[47] Yan X., Yao Y., Zhang H., Xie J., Xiao C., Zhang S., Qi J., Sun X., Li J. (2022). Zeolitic imidazolate framework (ZIF-8)/polyacrylonitrile derived millimeter-sized hierarchical porous carbon beads for peroxymonosulfate catalysis. Environ. Res..

[48] Zhong Q., Lin Q., Huang R., Fu H., Zhang X., Luo H., Xiao R. (2020). Oxidative degradation of tetracycline using persulfate activated by N and Cu codoped biochar. Chem. Eng. J..

[49] Wang H., Chen T., Chen D., Zou X., Li M., Huang F., Sun F., Wang C., Shu D., Liu H. (2020). Sulfurized oolitic hematite as a heterogeneous Fenton-like catalyst for tetracycline antibiotic degradation. Appl. Catal. B Environ..

[50] Ge L., Yue Y., Wang W., Tan F., Zhang S., Wang X., Qiao X., Wong P.K. (2021). Efficient degradation of tetracycline in wide pH range using MgNCN/MgO nanocomposites as novel H_2_O_2_ activator. Water Res..

[51] Hu Y., Chen D., Zhang R., Ding Y., Ren Z., Fu M., Cao X., Zeng G. (2021). Singlet oxygen-dominated activation of peroxymonosulfate by passion fruit shell derived biochar for catalytic degradation of tetracycline through a non-radical oxidation pathway. J. Hazard. Mater..

[52] Xiao G., Xu T., Faheem M., Xi Y., Zhou T., Moryani H.T., Bao J., Du J. (2021). Evolution of singlet oxygen by activating peroxydisulfate and peroxymonosulfate: A review. Int. J. Environ. Res..

[53] Nie M., Deng Y., Nie S., Yan C., Ding M., Dong W., Dai Y., Zhang Y. (2019). Simultaneous removal of bisphenol A and phosphate from water by peroxymonosulfate combined with calcium hydroxide. Chem. Eng. J..

[54] Cai H., Zou J., Lin J., Li J., Huang Y., Zhang S., Yuan B., Ma J. (2022). Sodium hydroxide-enhanced acetaminophen elimination in heat/peroxymonosulfate system: Production of singlet oxygen and hydroxyl radical. Chem. Eng. J..

[55] Wang J., Wang S. (2018). Activation of persulfate (PS) and peroxymonosulfate (PMS) and application for the degradation of emerging contaminants. Chem. Eng. J..

[56] Qi C., Liu X., Ma J., Lin C., Li X., Zhang H. (2016). Activation of peroxymonosulfate by base: Implications for the degradation of organic pollutants. Chemosphere.

