# Supplementary material for: Investigation into the Synergistic Effect of the Zinc Peroxide/Peroxymonosulfate Double-Oxidation System for the Efficient Degradation of Tetracycline
Source: Molecules. 2024 Aug 30;29(17):4120. doi: 10.3390/molecules29174120 (PMC11397340; doi:10.3390/molecules29174120)
Supplement: Supplementary file 1 [file molecules-29-04120-s001.zip › molecules-3159528-supplementary.pdf]

Supplementary Materials

Investigation into the Synergistic Effect of the Zinc Peroxide/Peroxymonosulfate Double-Oxidation System for the Efficient Degradation of Tetracycline

Shefeng Li [1,2,3], Yong Zhang [1,2,3], Siyu Ding [1,2,3], Xuli Li [1,2,3], Wei Wang [4], Ningning Dong [5], Miaomiao Nie [1,2,3] and Pei Chen [1,2,3,4,*]

1 School of Chemical and Environmental Engineering, Wuhan Polytechnic University, Wuhan 430023, China
2 Hubei Engineering Research Center for Soil and Groundwater Pollution Control, Wuhan 430070, China
3 Pilot Base of Ecological Environmental Chemicals and Low-Carbon Technology Transformation, Wuhan 430023, China
4 State Key Laboratory of Materials Processing and Die & Mould Technology, School of Materials Science and Engineering, Huazhong University of Science and Technology, Wuhan 430074, Hubei, China
5 Analytical and Testing Center, Huazhong University of Science and Technology, Wuhan 430074, China
* Correspondence: chenpei@whpu.edu.cn; Tel.: +86-27-83943956; Fax: +86-27-83943956

Text S1. HR-LC-MS analysis conditions and parameters

The specific sample pretreatment for HR-LC-MS test: At specific time intervals, 10 mL of mixture was extracted from degradation system and filtered through a 0.22-μm filter. Immediately, 0.1 mL of sodium thiosulfate solution (0.1 mol/L) was added into the above filtrate to terminate degradation process. The detailed HR-LC-MS instrumental analysis conditions and parameters were listed as follows:

LC conditions: the reverse-phase column (Accucore aQ, 100 × 2.1 mm, 2.6 um) was kept at 25°C; the eluent flow rate was 0.2 mL/min and the scanning range was 190-400 nm. LC separation was performed with a gradient elution program (Table R1) of methanol and formic acid aqueous solution (0.1 wt%) as mobile phase.

Table S1. Gradient elution program.

Time (min)	Methanol	Formic acid aqueous solution (0.1 wt%)
0	5%	95%
1	5%	95%
15	70%	30%
18	70%	30%
18.1	5%	95%
25	5%	95%

Mass condition: the ion source was ESI source with positive mode; the spray voltage was 3200 V; the max spray current was 100 μA; the ion transport capillary temperature was 300℃; the probe heater temperature was 300 ℃; the atomization and auxiliary gas were 40 arb and 8 arb, respectively.

Table S2. Crystallite sizes of ZnO_2 samples prepared under different agitation time.

Sample	FWHM	θ (°)	D (nm)
ZnO_2 (10 min)	0.964	36.81	9.13
ZnO_2 (30 min)	0.912	36.94	9.38
ZnO_2 (60 min)	0.803	36.97	10.59
ZnO_2 (240 min)	0.792	36.98	10.72

Text S2. Degradation kinetics analysis

The pseudo-zero order, pseudo-first order and pseudo-second order kinetics models (Eq. S1–S3) were employed to fit with the experimental data to illustrate the photocatalytic degradation process.

$$C_0 - C_t = k_0 t \quad \text{(Eq. S1)}$$

$$\ln\left(\frac{C_t}{C_0}\right) = -k_1 t \quad \text{(Eq. S2)}$$

$$\frac{1}{C_0} - \frac{1}{C_t} = -k_2 t \quad \text{(Eq. S3)}$$

Where, k_0 ($L \cdot mg^{-1}$), k_1 (min^{-1}) and k_2 ($L \cdot mg^{-1} \cdot min^{-1}$) were the rate constants of the pseudo-zero order, pseudo-first order and pseudo-second order kinetic model, respectively. C_0 (mg/L) was the concentration of TC at the initial time, while C_t (mg/L) was the concentration of TC at the certain time t (min). The rate constants of different models were directly calculated by slopes of linear equations, and the corresponding results were listed in Table S1. The fitting results of pseudo-zero order and pseudo-second order kinetic models were displayed in Fig. S1.

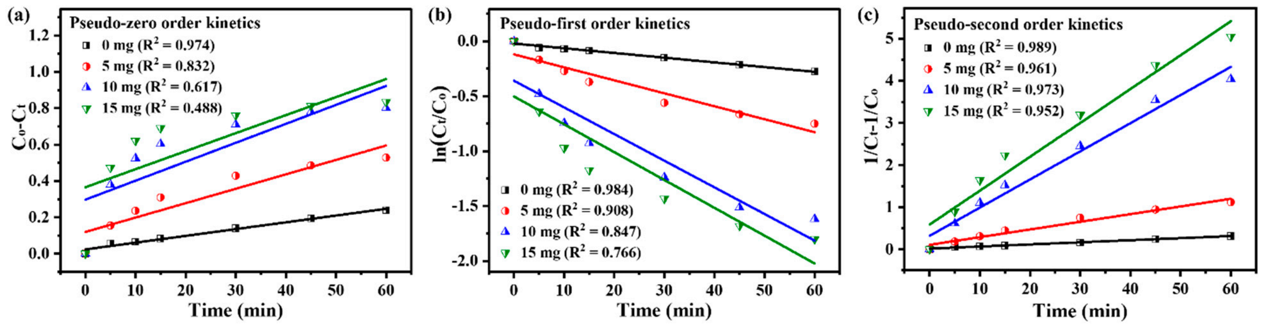

Figure S1. Linear curves fitted by pseudo-zero order (a), pseudo-first order (b) and pseudo-second order (c) kinetic models.

Table S3. Kinetic parameters of different kinetics models.

ZnO_2 dosage	Pseudo-zero order		Pseudo-first order		Pseudo-second order	
	k_0 ($L \cdot mg^{-1}$)	R_0^2	k_1 (min^{-1})	R_1^2	k_2 ($L \cdot mg^{-1} \cdot min^{-1}$)	R_2^2
0 mg	0.004	0.974	0.004	0.983	0.005	0.989
5 mg	0.008	0.832	0.012	0.908	0.018	0.961
10 mg	0.010	0.617	0.024	0.847	0.067	0.973
15 mg	0.010	0.488	0.025	0.766	0.080	0.952

Text S3. Degradation thermodynamics analysis

The Arrhenius equation (Eq. S4) was employed to investigate the thermodynamic feature of TC degradation in ZnO_2/PMS double-oxidation system.

$$k = A*\exp(-E_a/RT) \qquad \text{(Eq. S4)}$$

Where, k represented the rate constant, R was the molar gas constant, 8.314 $J\bullet mol^{-1}\bullet K^{-1}$. E_a was the apparent activation energy ($KJ\bullet mol^{-1}$), A was the pre-exponential factor, and T (K) was the reaction temperature.

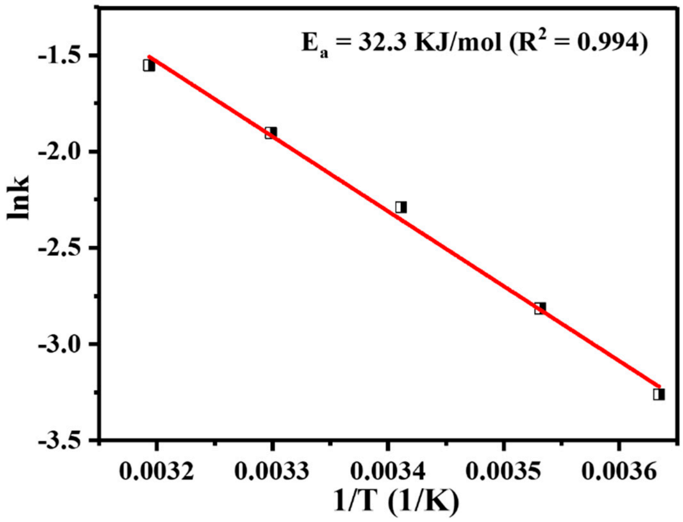

Figure S2. Linear curves fitted by Arrhenius equation.

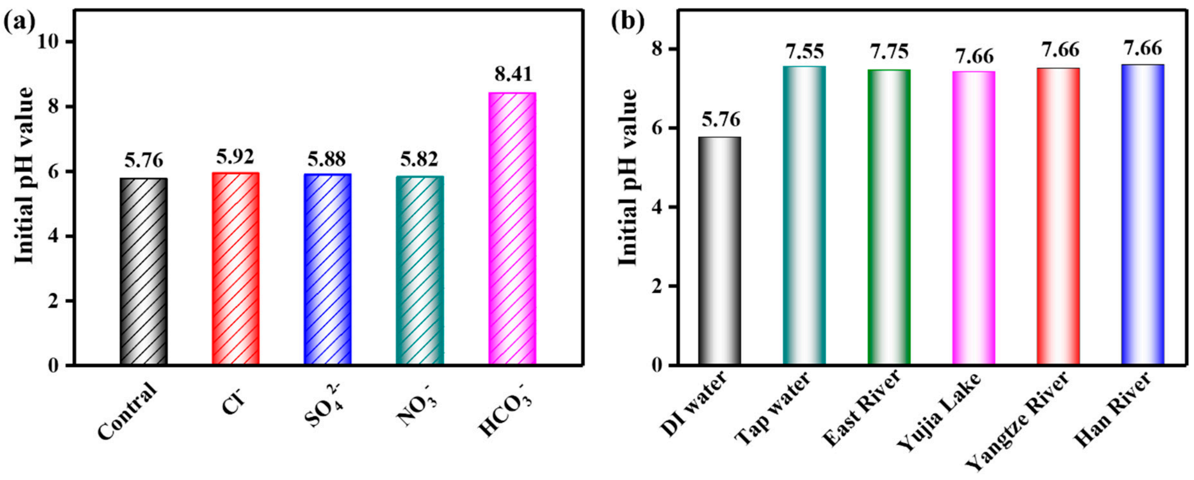

Figure S3. Initial pH values of TC solutions containing common inorganic anions (a) and prepared using different water sources (b).

Table S4. Water quality parameters of natural water resources.

Water resources	Water quality grade	Dissolved oxygen (mg/L)	COD (mg/L)	BOD_5 (mg/L)	NH_3-N (mg/L)	Total phosphorus (mg/L)	Total nitrogen (mg/L)	Fecal coliform (number/L)
Yangtze River	II	≥ 6	≤ 15	≤ 3	≤ 0.5	≤ 0.1	≤ 0.5	≤ 2000
Han River	II	≥ 6	≤ 15	≤ 3	≤ 0.5	≤ 0.1	≤ 0.5	≤ 2000
East Lake	III	≥ 5	≤ 20	≤ 4	≤ 1.0	≤ 0.2	≤ 1.0	≤ 10000
Yujia Lake	III	≥ 5	≤ 20	≤ 4	≤ 1.0	≤ 0.2	≤ 1.0	≤ 10000
Tap water	I (A)	≥ 7.5	≤ 3	≤ 3	≤ 0.5	≤ 0.1	≤ 0.5	0

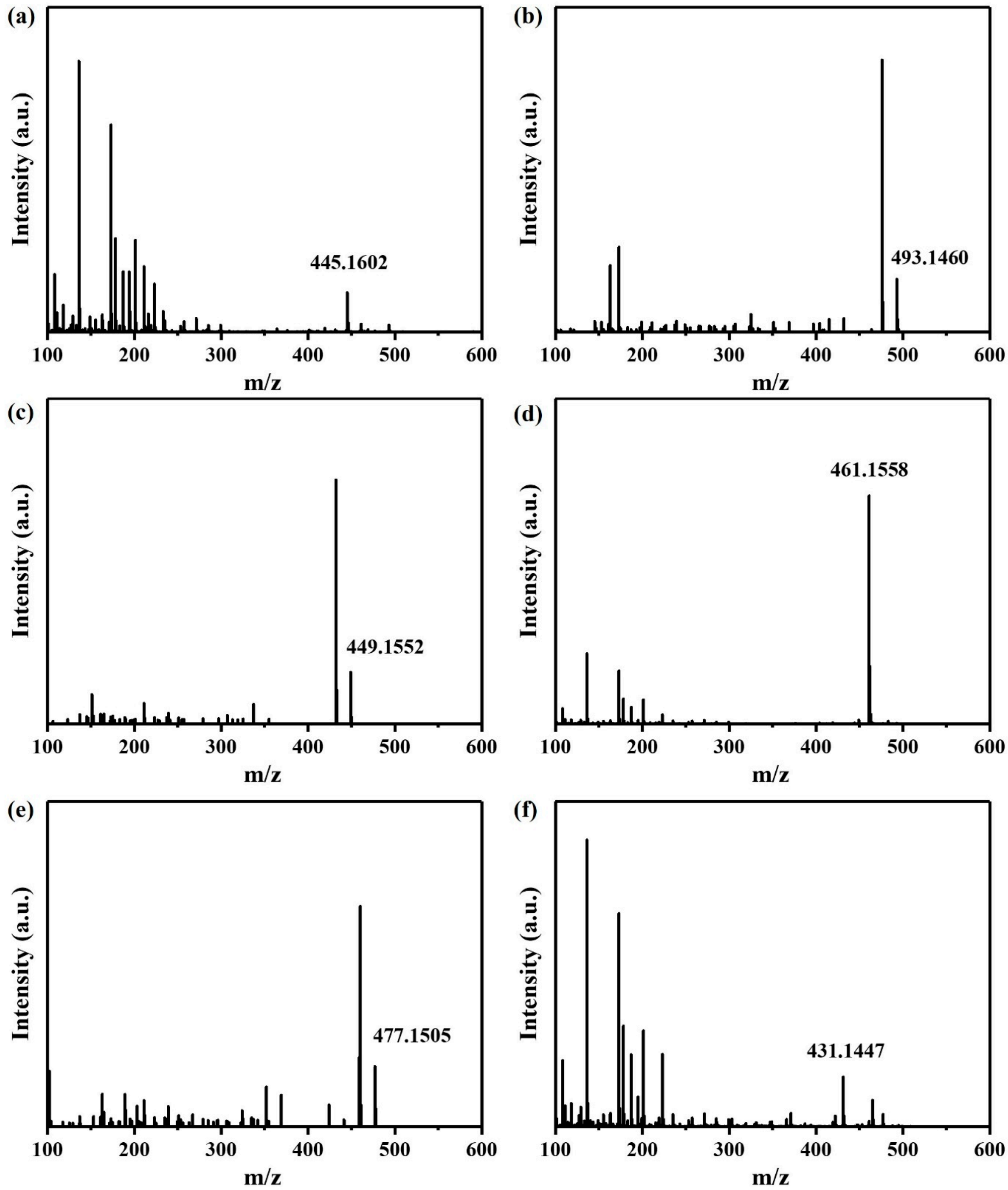
(a)
Intensity (a.u.)
445.1602
m/z
(b)
Intensity (a.u.)
493.1460
m/z
(c)
Intensity (a.u.)
449.1552
m/z
(d)
Intensity (a.u.)
461.1558
m/z
(e)
Intensity (a.u.)
477.1505
m/z
(f)
Intensity (a.u.)
431.1447
m/z
100
200
300
400
500
600

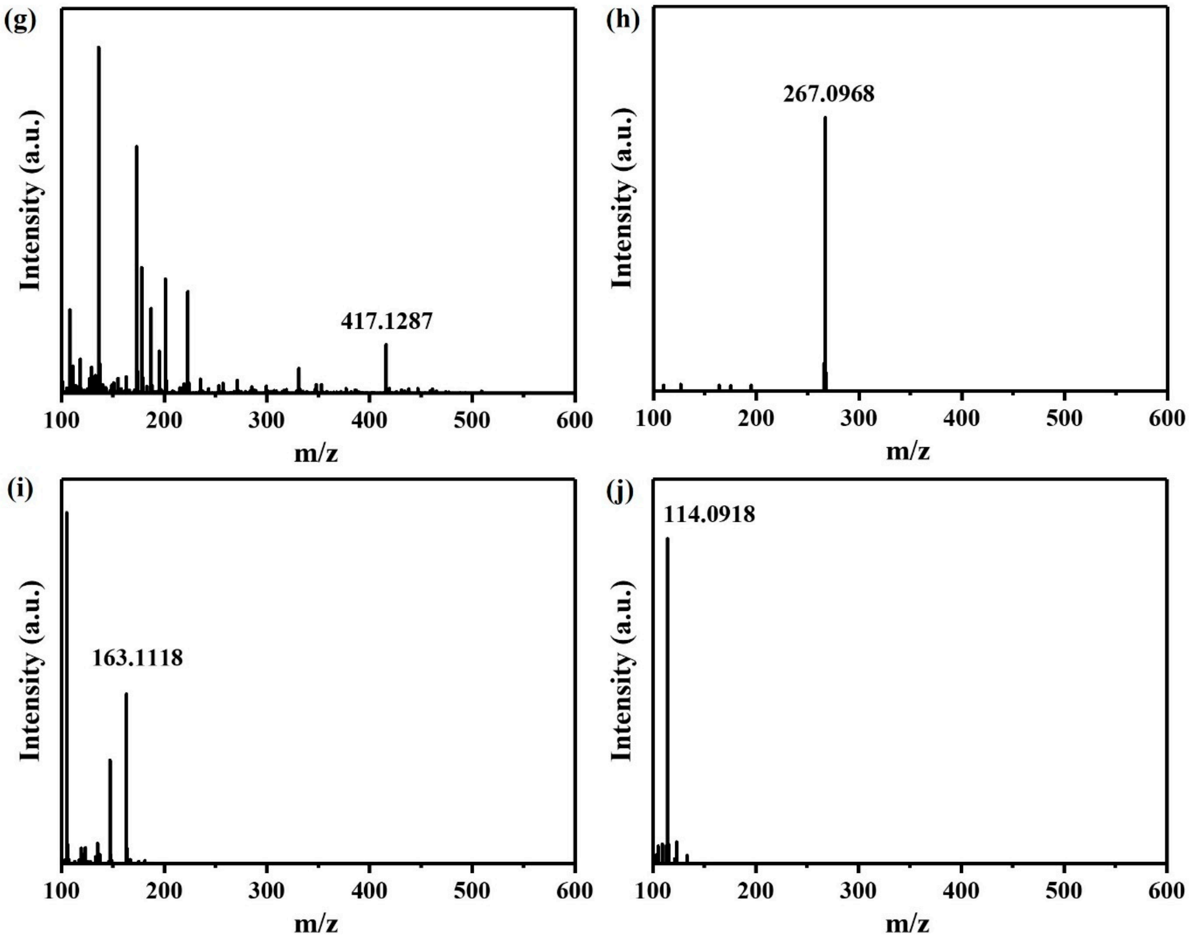

Figure S4. The detected mass spectra of TC and intermediates.

Table S5. Characteristics of intermediate products during TC degradation.

Abbreviation	Chemical formula (mode)	Theoretical m/z	Experimental m/z	Error (ppm)
P445	$C_{22}H_{25}N_2O_8$ ([M+H]+)	445.1605	445.1602	-0.72
P493	$C_{22}H_{25}N_2O_{11}$ ([M+H]+)	493.1453	493.1460	1.38
P449	$C_{21}H_{25}N_2O_9$ ([M+H]+)	449.1555	449.1552	-0.78
P461	$C_{22}H_{25}N_2O_9$ ([M+H]+)	461.1555	461.1558	0.65
P477	$C_{22}H_{25}N_2O_{10}$ ([M+H]+)	477.1504	477.1505	0.23
P431	$C_{21}H_{23}N_2O_8$ ([M+H]+)	431.1449	431.1447	-0.56
P417	$C_{20}H_{21}N_2O_8$ ([M+H]+)	417.1292	417.1287	-1.39
P267	$C_{12}H_{15}N_2O_5$ ([M+H]+)	267.0976	267.0968	-2.92
P163	$C_{11}H_{15}O$ ([M+H]+)	163.1117	163.1118	0.12
P114	$C_6H_{12}NO$ ([M+H]+)	114.0913	114.0918	3.68
